# MicroRNA-34a: A Novel Therapeutic Target in Fibrosis

**DOI:** 10.3389/fphys.2022.895242

**Published:** 2022-06-20

**Authors:** Min Zhao, Qin Qi, Shimin Liu, Rong Huang, Jiacheng Shen, Yi Zhu, Jing Chai, Handan Zheng, Huangan Wu, Huirong Liu

**Affiliations:** ^1^ Department of Acupuncture-Moxibustion, LongHua Hospital Shanghai University of Traditional Chinese Medicine, Shanghai University of Traditional Chinese Medicine, Shanghai, China; ^2^ Key Laboratory of Acupuncture and Immunological Effects, Shanghai University of Traditional Chinese Medicine, Shanghai, China; ^3^ Shanghai Research Institute of Acupuncture and Meridian, Shanghai, China

**Keywords:** microRNA-34a, fibrosis, apoptosis, autophagy, senescence, TGF-β1/Smad signal pathway, target genes

## Abstract

Fibrosis can occur in many organs, and severe cases leading to organ failure and death. No specific treatment for fibrosis so far. In recent years, microRNA-34a (miR-34a) has been found to play a role in fibrotic diseases. MiR-34a is involved in the apoptosis, autophagy and cellular senescence, also regulates TGF-β1/Smad signal pathway, and negatively regulates the expression of multiple target genes to affect the deposition of extracellular matrix and regulate the process of fibrosis. Some studies have explored the efficacy of miR-34a-targeted therapies for fibrotic diseases. Therefore, miR-34a has specific potential for the treatment of fibrosis. This article reviews the important roles of miR-34a in fibrosis and provides the possibility for miR-34a as a novel therapeutic target in fibrosis.

## Introduction

Fibrosis (FB) is an excessive repair reaction of the body to external injury, resulting in structural damage and dysfunction of normal tissues and organs, which affects the patients’ physical and mental health and quality of life seriously (Jun and Lau, 2018; Henderson et al., 2020). It is a high-burden diseases, and the annualized incidence of major fibrosis-related conditions is nearly 1/20 (Tsou et al., 2014; Zhao et al., 2020). At present, the treatment methods are limited. In the early stage, drug therapy merely alleviate inflammation and symptoms; in the late stage, only surgery or organ transplantation can be selected. However, the cure rate is still low and the recurrence rate is high (Rieder et al., 2012; Villac Adde et al., 2018; Ramos et al., 2019; Cai et al., 2020). Some researches has investigated a variety of regulator (such as microRNA, TGF-β, interleukins, IFN-γ) for the treatment of FB, which only a certain efficacy (Ghosh et al., 2013; Richeldi et al., 2017; Gieseck et al., 2018; Weiskirchen et al., 2019). As the signal transduction network of FB is complex, the current researches on therapeutic targets is not sufficient to support the clinical practice of FB. We need to further clarify the specific function of various signal molecules in fibrosis to guide the clinical therapy.

Recently, many studies have found that microRNA-34a (miR-34a) plays a role in a variety of fibrotic diseases by regulating cell proliferation, differentiation, apoptosis and other processes (Chen and Hu, 2012; Alivernini et al., 2014; Zhou et al., 2017; Li et al., 2018) (Table 1). It has been found that miR-34a can regulate the extracellular matrix (ECM) deposition by acting on the processes of apoptosis, senescence and autophagy in epithelial/endothelial cells and fibroblasts (Tian et al., 2016; Cui et al., 2017a; Zhu et al., 2019), and also promote transforming growth factor-β1 (TGF-β1)-induced fibroblasts activation by targeting Smad4 (Huang et al., 2014; Qi et al., 2020); while the miR-34a inhibitor can improve collagen deposition and attenuate fibrosis by regulating cell apoptosis and differentiation through Bcl-2, TGF-β1, and PPAR—γ(Zhou et al., 2014; Li et al., 2015; Song et al., 2019).

**TABLE 1:** MiR-34a acts on various organ fibrosis.

Tissue	Species	Target	Mechanism	References
Liver	Rat, hepatocyte, mice, intrahepatic biliary epithelial cells, HSCs, human	SIRT1, p53; caspase2	apoptosis	Tian et al. (2016), Meng et al. (2012)
		ACSL1; PPAR-γ; RXRa	target genes	Yan et al. (2015); Yan. (2016), Oda et al. (2014);Li et al. (2015)
		Smad4, Smad3	TGF-β1/Smad pathway	Feili et al. (2018), Song et al. (2019)
		p16、p21、CCL2、PAI-1	Cellular senescence	Wan et al. (2017)
Kidney	Mice, rat, renal tubular epithelial cells, renal interstitial fibroblasts	Bcl-2	apoptosis	Zhou et al. (2014); Li et al. (2019)
		Klotho; Notch1	target genes	Liu et al. (2019), Du et al. (2012)
		SIRT1	autophagy	Xue et al. (2018), Zhu et al. (2019)
Heart	Rat, myocardial fibroblasts, mice	C-Ski; PNUTS	target genes	Zhang et al. (2018), Boon et al. (2013)
		Smad4	TGF-β1/smad pathway	Huang et al. (2014)
		PI3K/AKT	autophagy	Liu et al. (2018)
Lung	Human, mice, type II alveolar epithelial cells	SIRT1, p53	apoptosis	Shetty et al. (2017)
		nectin-1、Abca3	target genes	Takano et al. (2017)
		E2F1、c-Myc、CCNE2	Celluar senescence	Disayabutr et al. (2016); Cui et al. (2017b)
Skin	mice	c-Met	target genes	Simone et al. (2014)

According to current researches, miR-34a may be exploited as a potential target for anti-fibrosis therapy in the future. In this paper, we review studies on the involvement of miR-34a in fibrotic diseases in order to reveal the possible mechanism of miR-34a as a therapeutic target for FB.

### Role of MicroRNA-34a in Various Molecular Pathways of Fibrosis

**FIGURE 1:**
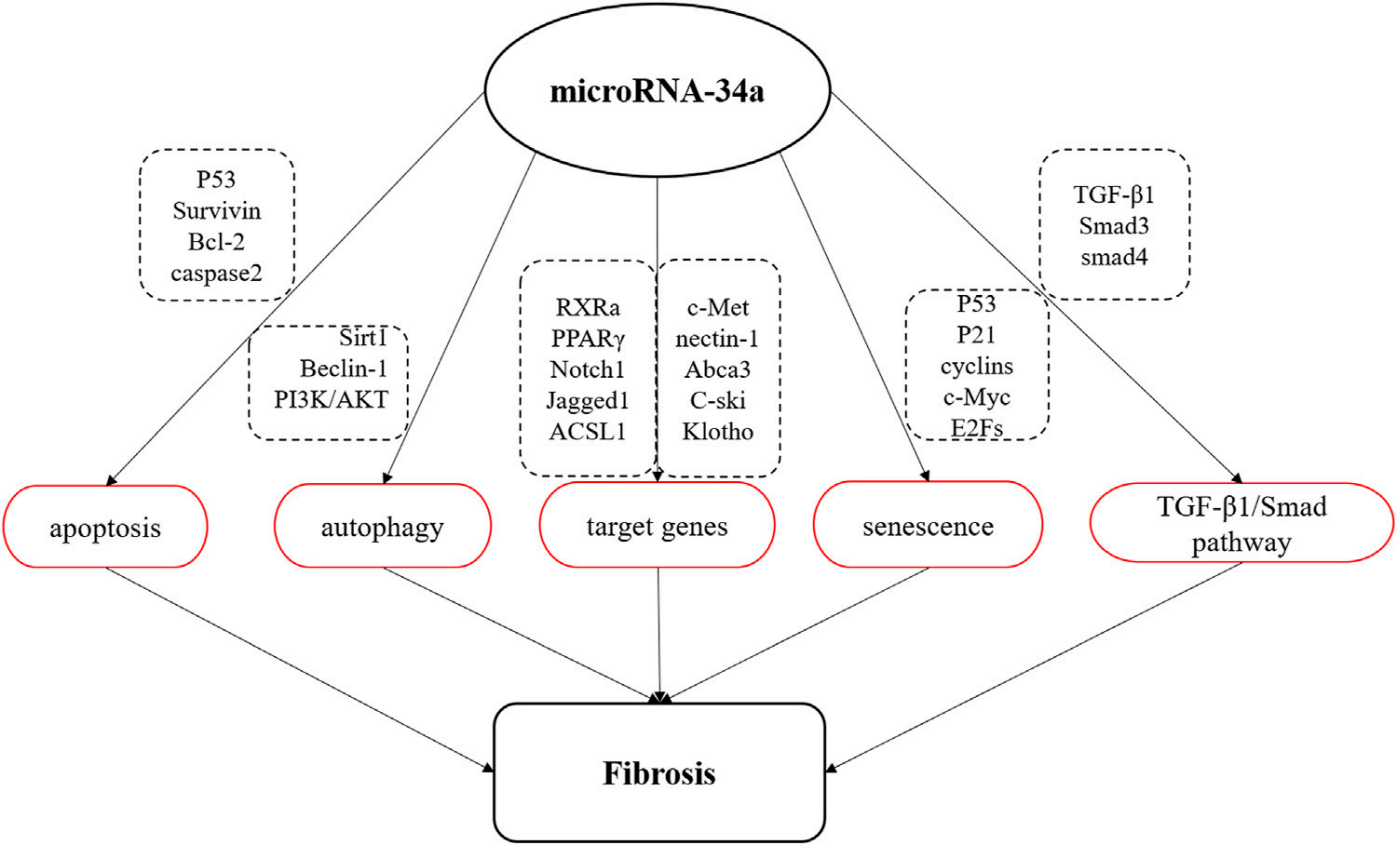
Schematic diagram of miR-34a involved in fibrosis process. MiR-34a is involved in the apoptosis, autophagy and senescence, regulates TGF-β1/Smad signal pathway, and negatively regulates the expression of multiple target genes to affect the process of organ fibrosis.

MicroRNA are a class of small non-coding RNA containing about 18–22 nucleotides that regulate gene expression at the post-transcriptional level through completely or partially complementary base binding to their target mRNAs (Tang et al., 2015). MiR-34a is a member of miRNA family, which is widely expressed in mammals (Hermeking, 2010). It has been found that miR-34a affects the occurrence and development of fibrotic diseases by regulating cell activities, including apoptosis, autophagy, cellular senescence, the expression of related target genes and TGF-β1/Smad signaling pathway (Figure 1).

### Role of MicroRNA-34a in Apoptosis

Apoptosis is a process of programmed cell death in multicellular organisms, which plays an important role in the development of fibrosis (Zhang et al., 2001; Docherty et al., 2006). After injury, cell apoptosis induce the recruitment of immune cells with amplification of inflammatory response and profibrogenic factors, enhance fibroblast proliferation, and then promotes the regeneration of granulation tissue, which eventually leads to the development of fibrotic lesions (Uhal, 2002; Bhandary et al., 2012; Jun and Lau, 2018). MiR-34a has been found to play an important role in the process of fibrosis by regulating apoptosis.

MiR-34a is the direct transcription target of p53, and can negatively regulate sirtuin 1(SIRT1), resulting in increased p53 acetylation. P53 and SIRT1 are typical genes involved in apoptosis regulation (Yamakuchi et al., 2008; Li et al., 2011), and p53 is a major contributor to the onset and progression of fibrotic diseases (Yang et al., 2010; Sutton et al., 2013; Overstreet et al., 2014; Valentijn et al., 2021; Fu et al., 2022; Li et al., 2022). Therefore, the miR-34a SIRT1/p53 signaling pathway forms a positive feedback loop that has a vital role in cell proliferation and apoptosis (Tarasov et al., 2007; Kumamoto et al., 2008; Kim et al., 2015). It was found that the expression level of miR-34a was positively correlated with the severity of liver injury (Castro et al., 2013). In liver tissue of rats with hepatic fibrosis, it has been observed that miR-34a and acetyl-p53 were up-regulated and SIRT1 was down-regulated; nevertheless, SIRT1 activator significantly reduced the levels of miR-34a and acetyl-p53, and inhibited fibrosis, which suggested that miR-34a/SIRT1/p53 signaling pathway was activated in fibrosis; *in vitro*, it was further confirmed that miR-34a/SIRT1/p53 signaling pathway was activated in epithelial cells to induce apoptosis, which activate hepatic stellate cells (HSCs) and accelerate the process of liver fibrosis (Tian et al., 2016). In addition, in the lung tissues of patients and mice with pulmonary fibrosis, the apoptosis levels of alveolar epithelial cells (AECs) were increased, the expression of acetyl-p53, PAI-1, and miR-34a was increased, and the expression of SIRT1 was decreased; however, the above process could be reversed by knockout of the miR-34a gene (Shetty et al., 2017). It can be seen that miR-34a/SIRT1/p53 is also involved in the apoptosis of pulmonary epithelial cells and the induction of pulmonary fibrosis.

Bcl-2 is an important antiapoptosis gene and one of the target genes of miR-34a. MiR-34a can promote apoptosis by inhibiting bcl-2 expression (Bommer et al., 2007). Tubular epithelial cells apoptosis is one of the mechanisms of tubular atrophy and tubulointerstitial fibrosis (Docherty et al., 2006). In the study of rats and mice with renal interstitial fibrosis, miR-34a was released from mesenchymal fibroblasts and transferred to proximal tubular epithelial cells, where it promoted apoptosis of renal tubular epithelial cells by inhibiting the transcription and translation of Bcl-2, further aggravating renal interstitial fibrosis (Zhou et al., 2014; Li et al., 2019).

Furthermore, caspase-2 is also the target gene of miR-34a, which helps to enhance apoptosis and plays a role in cell remodeling and tissue repair (Madesh et al., 2009). In the study of alcoholic liver disease, miR-34a was found to regulate apoptosis of hepatocytes and intrahepatic biliary epithelial cells by targeting caspase 2, affecting cell survival and migration, and regulating the release of matrix metalloproteinases (MMPs). Therefore, miR-34a plays a role in the repair of liver injury and liver fibrosis (Meng et al., 2012). The above results indicate that miR-34a participates in organ fibrosis by regulating apoptosis-related signal molecules.

### Role of MicroRNA-34a in Autophagy

Autophagy is a conserved lysosomal degradation process in eukaryotic cells that plays an important role in maintaining homeostasis in cells and tissues. Autophagy disorders participate in the development of organ fibrosis. It has been confirmed that autophagy promote the clearance of damaged proteins and organelles, and accelerate the degradation of extracellular matrix proteins (Ding and Choi, 2014; Lv et al., 2017; Jesus et al., 2019); in addition, intracellular autophagy flux can increases the energy needed for extracellular matrix protein formation (Kota et al., 2017). Some studies have found that autophagy mediates fibrotic diseases regulated by miR-34a.

A study of epidural scar hyperplasia after laminectomy has found that the expression of miR-34a and autophagy-related molecules (beclin-1, ATG5, LC3B-2/1, p53) were changed, which suggests that the disorder of miR-34a and autophagy level may be involved in the formation of fibrosis (Wang B. B. et al., 2017). The PI3K/Akt signaling pathway is a classical autophagy regulatory pathway involved in the regulation of cell proliferation, migration and differentiation (Zundler et al., 2016; Aoki and Fujishita, 2017; Shi et al., 2017). This signaling pathway is concerned in the study of myocardial fibrosis. In the rat model of myocardial fibrosis induced by thyroid hormone, miR-34a expression and PI3K and Akt proteins were found to be upregulated, while autophagy related proteins (ATG5, Atg7, Atg16L1, Beclin1, LC3A) were significantly downregulated, and MMPs/TIMPs ratios appeared imbalance. This study suggested that myocardial fibrosis might be related to miR-34a-mediated regulation of the PI3K/Akt signaling pathway and inhibition of autophagy (Liu et al., 2018).

In addition, miR-34a indirectly interferes with the extension of autolysosomes by inhibiting SIRT1(Yang et al., 2013). SIRT1 is not only a molecule involved in autophagy activation, but also an important component of the EMT, which plays an important role in the process of organ fibrosis (Salminen and Kaarniranta, 2009; Simic et al., 2013). It has been found that miR-34a-5p is up-regulated accompanied by the corresponding down-regulation of SIRT1 in the renal tissue of mice with diabetic nephropathy. MiR-34a-5p was positively correlated with the expression of fibronectin (FN), type I collagen (COL 1), and TGF-β1; then the cell experiments further identified that miR-34a-5p directly suppressed SIRT1 to increase the profibrogenic effects of TGF-β1 by targeting the 3′-UTR of SIRT1; it has also been found that miR-34a-5p inhibitor increases the expression of SIRT1 and decreases the level of TGF-β1, FN, and COL 1, then a small interfering RNA (siRNA) targeting SIRT1 enhanced the expression of TGF-β1 and FB-related genes, indicating that miR-34a-5p could promote renal fibrosis by inhibiting SIRT1 (Xue et al., 2018). In diabetic cardiomyopathy, miR-34a was also found to aggravate myocardial injury related to inhibition of SIRT1 transcription (Zhu et al., 2019). According to the current research, we found that miR-34a is involved in the fibrosis process by inhibiting autophagy-related molecules. Unfortunately, there is insufficient evidence to explore the role of miR-34a in fibrosis by regulating autophagy at present, further research is needed to fill in this theory in the future.

### Role of MicroRNA-34a in Cellular Senescence

Cellular senescence is a process in which cells undergo irreversible cell cycle arrest and is considered to play a key role in damage repair. Fibroblast senescence is one of the important factors of fibrosis pathology (Waters et al., 2018). It has been found that fibroblasts derived from fibrotic tissue have a variety of senescence-related characteristics. Myofibroblasts senescence stop synthesizing collagen and other ECM proteins, and secrete ECM protein-degrading enzymes to improve matrix deposition and limit the accumulation of fibrotic tissue (Harding et al., 2005; Krizhanovsky et al., 2008; Jun and Lau, 2010; Álvarez et al., 2017). Besides, epithelial cells senescence indirectly promotes the differentiation of fibroblasts into myofibroblasts, resulting in the excessive deposition of collage (Lehmann et al., 2017).

As a downstream transcription target of p53, a cell cycle regulator, miR-34a is closely related to cell senescence (Kyle et al., 2009; Harries, 2014). It has been proved that miR-34a can regulate cell cycle and senescence by targeting multiple genes, such as SIRT1, cyclin E2, cyclin D1, and E2F3 (Cui et al., 2017a). AECs are the main senescent cells of pulmonary fibrosis. In the lung tissues and purified AECs of patients with idiopathic pulmonary FB (IPF), the relative levels of miR-34a, miR-34b and miR-34c were significantly increased, the activity of p16, p21, p53, and SA-β-gal was increased, and the expression of miR-34 targets (E2F1, c-myc, and CCNE2) was downregulated, these changes stimulated the senescence of AECs, promoted myofibroblast transdifferentiation and induced IPF (Disayabutr et al., 2016; Cui et al., 2017b). In the study of hepatic fibrosis, the same results were obtained. MiR-34a was up-regulated in the patients with hepatic fibrosis, which promoting the senescence of hepatocytes and inducing hepatic fibrosis by reducing the senescence of HSCs; however, miR-34a inhibitor (morpholino) obstructed this process and improved hepatic fibrosis, which indicating that miR-34a plays a role in promoting hepatocytes senescence and reducing HSCs senescence (Wan et al., 2017). Not only can miR-34a regulates epithelial cell senescence and induce fibroblast to differentiate into myofibroblast, but also inhibits fibroblast senescence, promotes fibroblast proliferation, and aggravates the fibrosis process. Therefore, cell senescence plays an important role in the process of miR-34a participating in fibrosis.

### Regulation MicroRNA-34a on Typical Target Genes

MiRNA-34a regulates growth, differentiation and metabolism by negatively regulating typical target genes. Previous studies have revealed that miR-34a can combined with multiple target genes to regulate fibrosis in many ways.

ACSL1 is a member of Acyl-CoA synthetase long-chain (ACSL) family. ACSL1 is an important gene in liver lipid metabolism. The luciferase reporter assay confirmed that ACSL1 was the target gene of miR-34a (Li et al., 2011). In the research of hepatic fibrosis, miR-34a specifically bound to the 3′-UTR of ACSL1, which negatively regulated the expression of ACSL1 mRNA and protein, promoted the activation and proliferation of HSCs, and lead to upregulation of ECM-related indicators (COL 1, a-SMA); in contrast, silencing of the miR-34a gene increased the expression of ACSL1, decreased the expression of ECM-related proteins, and affected HSCs activation (Yan et al., 2015; Yan, 2016). Thus, ACSL1 is one of the factors by which miR-34a promotes hepatic fibrosis.

Protooncogene c-ski, a transcriptional corepressor, is a negative regulator of TGF-β/Smad signaling (Cunnington et al., 2009), and can inhibit TGF-β1-induced activation of cardiac fibroblasts and ECM deposition (Wang J. et al., 2017). *In vitro* and *in vivo* studies on myocardial fibrosis in rats, it was found that miR-34a could target and inhibit the expression of c-ski, and the levels of collagen I and *a*—SMA were significantly increased; Inhibition of miR-34a significantly increased the expression of c-ski protein and decreased the levels of COL one and *a*-SMA protein (Zhang et al., 2018). It can be seen c-ski mediates miR-34a to promote the proliferation and ECM deposition of TGF-β1-induced primary cultured rat cardiac fibroblasts, which contribute to myocardial fibrosis.

Klotho, a specific antiaging protein of kidney, is mainly expressed in renal tubular epithelial cells and has a significant anti-fibrosis effect (Guan et al., 2014; Ding et al., 2019). The luciferase reporter assay showed that miR-34a directly down-regulated the expression of Klotho. In renal fibrosis, the increased expression of miR-34a is accompanied by the sharp downregulation of Klotho, the increase of *a—*SMA and fibronectin, and the decrease of E-cadherin, which promote the process of epithelial mesenchymal transformation (EMT); however, the expression of Klotho was significantly increased and EMT was inhibited in miR-34a−/− mice, so miR-34a negatively regulates Klotho to promote EMT and induce renal fibrosis (Liu et al., 2019).

In addition, there were other miR-34a target genes, including PPAR-γ, PNUTS, RXRa, Notch1, c-Met, nectin-1, and Abca3, have been found to affect the fibrosis process by regulating cell proliferation, the EMT process and collagen synthesis (Du et al., 2012; Boon et al., 2013; Oda et al., 2014; Simone et al., 2014; Li et al., 2015; Takano et al., 2017). In various organ fibrosis, miR-34a affects the process of fibrosis by targeting different protein-coding genes.

### Role of MicroRNA-34a in Transforming Growth Factor-β1/Smad Signaling Pathway

Transforming growth factor-β1 (TGF-β1) is a key cytokine involved in the formation of fibrosis (Ghosh et al., 2013) that not only plays an important role in the transdifferentiation of fibroblasts into myofibroblasts but also triggers the EMT, mesothelial-to-mesenchymal transition (MMT) and endothelial-to-mesenchymal-transition (EndoMT) processes, controls the extracellular matrix (ECM) synthesis, and participates in the pathogenesis of fibrosis (Wu et al., 2013; Weiskirchen et al., 2019). There is a certain correlation between miR-34a disorders and TGF-β pathway in fibrotic diseases (Xie et al., 2011; Zhang et al., 2014; Zhang J. et al., 2021).

Firstly, Bin Zhou found that eight miRNAs and seven mRNA were involved in TGF-β signal pathway, including miR-34a, in systemic sclerosis (SSc) by Gene Expression Omnibus (GEO) analysis (Zhou et al., 2017), this was a direct evidence that miR-34a targets fibrosis through TGF-β signaling pathway. Smad transcription factors are the core of TGF-β pathway (Massague et al., 2005). TGF-β1/Smad signaling pathway has been widely recognized as a typical pathway in fibrosis (Zhang et al., 2019; Lv et al., 2020). The expression of miR-34a was increased in mice with cardiac fibrosis, and the degree of fibrosis was inhibited by miR-34a antagonist; miR-34a directly targets Smad4 mRNA according to luciferase reporter assay; when the fibroblasts are transfected with Smad4 siRNA, the expression of type I collagen, TGF-β1 and *a*-SMA was suppressed. The study indicated that TGF-β1 induces the expression of miR-34a, which in turn promotes the activation of TGF-β1-induced myocardial fibroblasts and the formation of cardiac fibrosis by targeting Smad4 (Huang et al., 2014). In carbon tetrachloride (CCl4)-induced hepatic fibrosis mice, miR-34a imbalance was also found to promote liver fibrosis *via* targeting Smad4 and activation TGF-β1/Smad3 pathway (Feili et al., 2018).

Besides, miR-34a/SIRT1/p53 loop is also involved in the EMT mediated by TGF-β1/Smad signaling pathway. Activated p53 (ac-p53 and p-p53) combines with Smad3 to form a multiprotein complex to promote TGF-β1-induced EMT process (Piccolo, 2008; Termén et al., 2013). In rat model of hepatic fibrosis, it was found that miR-34a was overexpressed, SIRT1 was down-regulated, p53 and ac-p53 were increased, with activated TGF-β1/Smad signal pathway; miR-34a inhibitor and p53 siRNA significantly prevented TGF-β1-induced EMT in hepatocytes, and alleviated the degree of hepatic fibrosis (Song et al., 2019). Therefore, these results suggest that TGF-β1/Smad signaling pathway mediates the process of miR-34a-induced fibrosis.

## MiRNA-34a as Therapeutic Targets of Fibrosis

As described, miR-34a is a key regulator of FB-related molecules. In recent years, miR-34a or miR-34a-targeted gene have been used as new intervention targets in the treatment of FB, which have better effectiveness. Therefore, the regulation of miR-34a and related molecules are expected to be new therapeutic targets for FB (Table 2).

**TABLE 2:** the biological agents of miR-34a and related molecules for fibrosis.

Type	Biologics	Target	Tissue/Cell	References
miR-34a inhibitor	MiR-34a inhibitor	miR-34a	renal tubular epithelial cells, intrahepatic biliary epithelial cells, HSCs, hepatocyte, Cardiac fibroblasts, heart, liver, lung	Zhou et al. (2014), Yan et al. (2015), Li et al. (2015), Kim et al. (2015), Huang et al. (2014), Bernardo et al. (2016), Pan et al. (2021), Zhang et al. (2021a), Zhang et al. (2021b), Cui et al. (2017b)
	Hydrogen sulfide (H_2_S)		heart	Liu et al. (2018)
	Astragaloside-IV(AS-IVA)		cardiomyocytes	Zhu et al. (2019)
	Aqueous extract from Prunella Vulgaris (PVAE)		HSCs	Hu et al. (2016)
	Pterostilbene		hepatocyte	Song et al. (2019)
	Atorvastatin		endothelial cell	Tabuchi et al. (2012)
Preparation of miR-34a-related molecules	SRT1720	SIRT1	hepatocyte	Tian et al. (2016)
	Resveratrol	SIRT1	liver, kidney	Chávez et al. (2008); Hong et al. (2010); Li et al. (2010)
	pifithrin -α	p53	hepatocyte	Kim et al. (2015)
	PPARγ agonist	PPARγ	HSCs	Attia et al. (2013); Sharvit et al. (2013)
	Smad4 siRNA	Smad4	cardiac fibroblast	Huang et al. (2014)
	Jagged1 siRNAs	Jagged1	renal tubular epithelial cells	Du et al. (2012)
	Notch1siRNAs	Notch1	renal tubular epithelial cells	Du et al. (2012)
	LGR4 siRNA	LGR4	retinal pigment epithelial cells	Hou et al. (2016)
	PNUTS	PNUTS	heart	Boon et al. (2013)

### MicroRNA-34a Inhibitors

In most studies, miR-34a inhibitors were used to improve the degree of fibrosis. At the cellular level, miR-34a inhibitor was transfected into renal tubular cells incubated with TGF-β1 to induce the upregulation of Bcl-2, inhibit the apoptosis of renal tubular cells and improve the degree of renal fibrosis (Zhou et al., 2014). Transfection of miR-34a silencing vector using Lipofectamine2000 into activated HSCs increased the expression of ACSL1 and promoted lipogenesis, thereby inhibiting HSCs activation and hepatic fibrosis (Yan et al., 2015). MiR-34a inhibitor was also found to increase PPAR γ, decrease *a*-SMA, and improve the process of liver fibrosis (Li et al., 2015). Transfection of miR-34a inhibitor in primary hepatocytes increased SIRT1 and p65/p53 deacetylation levels, decreased the expression of proinflammatory cytokines and improved liver inflammatory response (Kim et al., 2015). MiR-34a inhibitor could reduce the EMT process and fibrosis activity of human intrahepatic biliary epithelial cells, and improved liver fibrosis (Pan et al., 2021). In cardiac fibroblasts, a miR-34a antagonist improved cardiac fibrosis by inhibiting TGF-β1 signaling (Huang et al., 2014). *In vivo* study, Subcutaneous injection of locked nucleic acid (LNA)-antimiR-34a (initial dose 25 mg/kg, maintenance dose 10 mg/kg every other day, 3 times a week for 6 weeks) can improved the cardiac function of female mice with dilated cardiomyopathy, characterized by attenuated heart enlargement and lung congestion, inhibit the expression of cardiac stress genes, and alleviate myocardial fibrosis (Bernardo et al., 2016). Besides, miR-34a inhibitors can improve myocardial fibrosis and reduce scar area in myocardial infarction rats (Zhang F. et al., 2021). In the mice of CCl4-induced liver fibrosis, miR-34a siRNA significantly reduced the express of TGF-β, *a*-SMA, and MCP-1, further inhibited the fibrosis of HSCs (Zhang J. et al., 2021). It has also been found that ablation of miR-34a protected aged animals from developing experimental lung fibrosis (Cui et al., 2017b).

In addition, there are some compounds acting on miR-34a to intervene in FB. Hydrogen sulfide and astragaloside IV(AS-IV) were found to reverse myocardial fibrosis, which may be related to the down-regulation of miR-34a to activate autophagy (Liu et al., 2018; Zhu et al., 2019). *Prunella vulgaris* aqueous extract (PVAE) can downregulate miR-34a level, inhibit the activation of HSCs, and regulate the expression of TIMP-1, MMP-2, and MMP-13, promoting the degradation of collagen, and alleviating hepatic fibrosis (Hu et al., 2016); Paclitaxel has been applied to treat fibrosis by downregulating miR-34a, upregulating SIRT1, and inhibiting p53 activation and TGF-β1/Smads signal pathway (Song et al., 2019). Atorvastatin also inhibited miR-34a and upregulated SIRT1 to improve myocardial fibrosis (Tabuchi et al., 2012). Therefore, the above study shows that the downregulation of miR-34a has therapeutic effect on FB.

### The Biological Agents of MicroRNA-34a-Related Molecules

The target gene of miR-34a has been used as the therapeutic target for fibrosis in some researches. SRT1720, the SIRT1activator, inhibited hepatocyte apoptosis and improved liver fibrosis by reducing the expression of miR-34a and the acylation of p53 (Tian et al., 2016). Resveratrol, another SIRT1 activator, was often used as an inhibitor in fibrosis researches (Chávez et al., 2008; Hong et al., 2010; Li et al., 2010). P53 inhibitor, pifithrin—*a* (PFT), decreased the level of miR-34a and played a protective role in hepatic ischemia/reperfusion mice (Kim et al., 2015). In addition, PPAR γ activators blocked the activation of HSCs in hepatic fibrosis (Attia et al., 2013; Sharvit et al., 2013). Smad4 siRNA downregulated the mRNA and protein expression of Col I, a-SMA, and TGF-β1, and inhibited myocardial fibrosis (Huang et al., 2014). Jagged1 siRNA and Notch 1 siRNAs effectively inhibited EMT in renal tubular epithelial cells (Du et al., 2012). LGR4 is the direct target of miR-34a, LGR4 siRNA significantly inhibited the proliferation and migration of retinal pigmented epithelial cell line ARPE-19 (Hou et al., 2016). As a novel direct miR-34a target, PNUTS improved the functional recovery after acute myocardial infarction by reducing telomere shortening, DNA damage response and cardiomyocyte apoptosis (Boon et al., 2013). These results suggest that miR-34a-related molecules also plays an important role in the treatment of FB, which may provide guiding significance for clinical research.

## Limitation of MicroRNA-34a as Therapeutic Targets of Fibrosis

Currently there are no FDA-approved miRNAs, but many miRNA therapies have achieved substantial preclinical efficacy, even entered in clinical trials (Wang et al., 2021; Smith et al., 2022; Zogg et al., 2022). For example, miravirsen (miR-122 inhibitor) has completed Phase II clinical trials for the treatment of Hepatitis C (Janssen et al., 2013; Panigrahi et al., 2022). The Phase I clinical trials of MRG-110 (miR-92a inhibitor) to improve wound healing has been completed (Gallant-Behm et al., 2018; Abplanalp et al., 2020). A Phase I/IIa clinical trial has demonstrated the potential of RG-125 (AZD4076) (miR-103/107 inhibitor) for the treatment of type 2 diabetes and non-alcoholic fatty liver disease (Rottiers and Naar, 2012). A Phase 1b clinical trial of RGLS4326 (miR-17 inhibitor) in patients with autosomal dominant polycystic kidney disease is under way (Kim and Park, 2016). A Phase I clinical trials have shown that CDR132L inhibits miR-132 in patients with heart failure (Ucar et al., 2012). Moreover, TargomiRs, a miR-16 mimic, has been considered as a second- or third-line treatment for recurrent malignant pleural mesothelioma and non-small cell lung cancer (van Zandwijk et al., 2017). Therefore, the therapeutic potential of miRNAs is limitless.

Based on the existing research, miR-34a plays a complex and important role in fibrotic diseases. It will be a new target for the treatment of FB, but there are still many practical problems for miR-34a as a therapeutic target. At present, the anti-fibrosis effect of miR-34a and its target molecules have been explored mainly at the cellular level *in vitro*, perhaps because of the functional complexity of miR-34a and the non-target effect *in vivo*. There are still some problems in the preparation of miR-34a inhibitors. Although liposome transfection has been used in some experiments, it has the disadvantage of immunogenicity (Simone et al., 2014; Yan et al., 2015). Viral delivery enables long-term, persistent, and high expression of miRNAs, but it also has the disadvantage of nonspecific binding, so it cannot transport miRNAs to the designated site. Microvesicles, a new cell signaling vector for short- or long-range delivery, contains protein, mRNA and miRNA (Martins et al., 2013; Recep et al., 2017). In a study of renal fibrosis, it has been found that (Zhou et al., 2014; Li et al., 2019) renal interstitial fibroblasts can secrete microvesicles containing miR-34a to transport to renal tubular epithelial cells and promote their apoptosis; then the microbubbles in fibroblasts can be extracted and injected into cells or mice to imitate the mechanism of miR-34a in renal fibrosis. With the development of science and technology, other better biological agents will likely be found in the future, further improving the treatment of FB.

## Conclusion

In summary, although there have been many studies on the pathogenesis of fibrosis, there are still many deficiencies in the treatment of fibrosis. Various types of fibrosis, such as pulmonary fibrosis, cardiac fibrosis, liver fibrosis, renal fibrosis, etc., involve the same or different internal signal network. Therefore, it is very difficult to find the common target of fibrosis. Although there were two drugs (pirfenidone and nintedanib) have been approved for the treatment of idiopathic pulmonary fibrosis, they can only improve lung capacity and survival rate, and do not show beneficial histological changes in pulmonary fibrosis (Martinez et al., 2017). Therefore, it is urgent to develop new anti-fibrosis therapy for other fibrotic diseases. MiR-34a can regulate the expression of many genes and proteins, and participate in complex signal mechanism. Compared with traditional cytokines and signal molecules, miR-34a is more suitable as a common target for the regulation of organ FB. MiR-34a is a key regulator of fibrosis, which is involved in the regulation of apoptosis, senescence, autophagy, and TGF-β1 signaling pathway in epithelial cells and fibroblasts to affect the excessive repair; moreover, target genes of miR-34a also regulate the process of fibrosis in many ways; the application of miR-34a inhibitor has also been found to significantly improve the degree of fibrosis. So miR-34a is expected to become a new target for the treatment of fibrosis. However, those vivo or clinical studies on the treatment of fibrosis with miR-34a are still little and incomplete, so the specific mechanism and efficacy need to be further verified.
